# Genetic variants and clinical determinants affecting the response to 5-Fluorouracil-based treatment in Chilean patients with advanced colorectal cancer

**DOI:** 10.3389/fonc.2025.1589724

**Published:** 2025-07-25

**Authors:** Leslie C. Cerpa, Christopher Sandoval, Paula Escalante, Juan P. Cayún, María A. Lavanderos, Claudio Alarcón-Concha, Guillermo Kaempfe, Diego Moreno-Tapia, Camilo S. Quiroz, Carolina Gutierrez-Cáceres, Olga Barajas, Bettina Müller, Alicia Colombo, Gerardo Donoso, Angie Nuñez, Nelson M. Varela, Luis A. Quiñones

**Affiliations:** ^1^ Laboratory of Chemical Carcinogenesis and Pharmacogenetics, Department of Basic-Clinical Oncology (DOBC), Faculty of Medicine, University of Chile, Santiago, Chile; ^2^ Latin American Network for Implementation and Validation of Clinical Pharmacogenomics Guidelines (RELIVAF), Santiago, Chile; ^3^ Center for Cancer Prevention and Control (CECAN), Santiago, Chile; ^4^ Clinical Laboratory, Felix Bulnes Clinical Hospital, Santiago, Chile; ^5^ Department of Basic Sciences, Faculty of Sciences, University of Bío, Bío, Chile; ^6^ Escuela de Medicina, Fundación Instituto Profesional Duoc UC, Santiago, Chile; ^7^ Department of Pharmaceutical Sciences and Technology, Faculty of Chemical and Pharmaceutical Sciences, University of Chile, Santiago, Chile; ^8^ Clinical Hospital of University of Chile, Santiago, Chile; ^9^ National Cancer Institute, Santiago, Chile; ^10^ Biobank of Fluids and Tissues of the University of Chile, Santiago, Chile; ^11^ Faculty of Pharmacy, University of Costa Rica, San Jose, Costa Rica

**Keywords:** colorectal cancer, 5-FU, pharmacogenetics, pharmacogenomics, adverse drug reactions

## Abstract

**Background:**

Colorectal cancer is the second most prevalent cancer in Chile, affecting both sexes. Late-stage diagnosis occurs in approximately 25% of cases, with a five-year survival rate of only 14%. Standard treatment involves surgical resection followed by 5-fluorouracil-based chemotherapy, often combined with oxaliplatin or irinotecan. However, patient responses vary significantly due to genetic polymorphisms affecting drug metabolism, including variants in TYMS, DPYD, GSTs, and DNA repair enzymes. While genetic factors influencing chemotherapy outcomes have been studied, their impact remains unclear and varies across populations. No predictive model integrating genetic and clinical variables for chemotherapy safety in Chilean colorectal cancer patients has been established.

**Objective:**

This study aimed to identify relevant genetic variants in *TYMS*, *TYMP*, *DPYD*, *GSTP1*, *MTHFR*, *ERCC2*, *ABCB1*, *ABCC2*, *ABCC4*, and *ABCG2* genes, which, combined with clinical factors, could contribute to a predictive model for 5-FU-based chemotherapy safety in advanced colorectal cancer patients.

**Methods:**

A retrospective nested case-control study was conducted on 82 advanced colorectal cancer patients. Sixteen genetic variants were analyzed to assess their association with adverse reactions and their severity using logistic regression. Multivariate models were developed to predict chemotherapy safety.

**Results:**

Among the 16 variants analyzed in 82 patients, key findings included: The G allele of *GSTP1* (rs1695) was protective against neuropathy (OR = 0.147; p = 0.012) but increased mucositis risk (OR = 2.27; p = 0.036). The C allele of *DPYD* (rs1801265) was linked to a higher neuropathy risk (OR = 4.58; p = 0.05). The *TYMS* deletion genotype (rs11280056) conferred protection against hematological adverse reactions (OR = 0.029; p = 0.001). On the other hand, the 3R genotype of *TYMS* 5’UTR (rs45445694) is associated as a risk factor for skin and subcutaneous tissue disorders (OR = 6.40; p = 0.029). Two multivariate models were developed to predict anemia (p = 0.027) and pain (p = 0.01) development.

**Conclusions:**

This study provides a foundation for developing pharmacogenetic-based predictive models for adverse reactions associated with 5-FU, including neuropathy, mucositis, and hematological and skin toxicities. Future research may refine these models to enable personalized dose adjustments, improving chemotherapy safety in Chilean colorectal patients.

## 1 Introduction

Colorectal cancer (CRC) is the most prevalent gastrointestinal malignancy in both Chile and worldwide, according to GLOBOCAN’s latest reports (1, 2). Fluoropyrimidine-based chemotherapy is the cornerstone of first-line treatment for colorectal cancer in Chile, particularly for patients diagnosed with advanced disease (3). These regimens typically combine a fluoropyrimidine (FP), such as 5-fluorouracil (5-FU) with leucovorin (LV) or its prodrug capecitabine alongside one or more cytotoxic agents. The most commonly used combinations include irinotecan, a topoisomerase-I inhibitor, and oxaliplatin (L-OHP), a platinum-based antineoplastic drug (4–7). Standard treatment protocols such as FOLFOX (5-FU + LV + L-OHP), CAPOX (capecitabine + L-OHP), and FOLFIRI (5-FU + LV + irinotecan) are preferred in first- and second-line therapy. In recent years, targeted therapies, including EGFR inhibitors (cetuximab, panitumumab) and the VEGF inhibitor bevacizumab have been gradually integrated into treatment strategies for CRC management in Chile (3).

Nonetheless, CRC chemotherapy regimens are not exempt from adverse drug reactions (ADR), which can range from mild to severe, potentially affecting treatment adherence, quality of life and overall survival (8). This is particularly critical for patients receiving L-OHP-containing regimens, where cumulative toxicity necessitates intermittent rather than continuous treatment (6, 7). A meta-analysis shows that at least 45,7% of patients experience moderate to severe ADRs-primarily gastrointestinal, neurological, and hematological-though underreporting in clinical practice suggests this figure may be even higher (8). Identifying biomarkers associated with ADR risk could serve as a valuable tool for predicting, preventing and managing toxicity, ultimately optimizing the safety and efficacy of FP-based chemotherapy.

**Figure 1:**
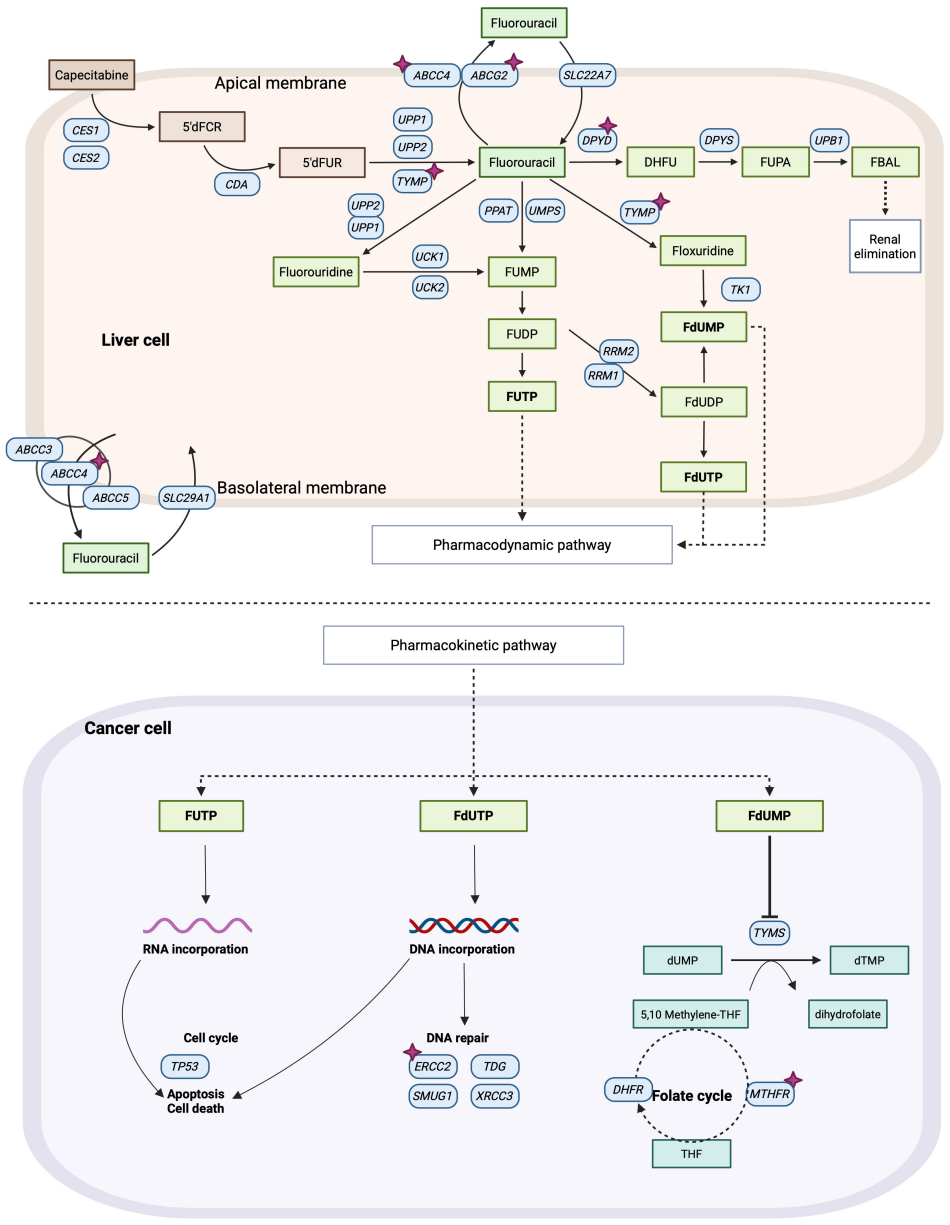
Pharmacokinetics and pharmacodynamics pathways of 5-Fluorouracil (5-FU). Purple stars indicate analyzed genes in this study. Image created with BioRender.com, adapted from PharmGKB.

Pharmacogenomic research has revealed that a myriad of proteins is involved in the pharmacokinetics and pharmacodynamics of 5-FU and L-OHP. Genetic variability within a population can lead to alterations in the genes encoding these proteins affecting individual responses to FP-based chemotherapy (9–12). Dihydropyrimidine dehydrogenase (DPD) is the primary enzyme responsible for 5-FU biotransformation and inactivation (**Figure 1**) is encoded by the highly polymorphic *DPYD* gene. Certain *DPYD* polymorphisms (DPYD*2A - rs3918290, c.1679T>G - rs55886062, c.2846A>T - rs67376798 or c.1236G>A - rs56038477) result in a complete loss of DPD function and are strongly associated with severe FP-related toxicity. Given their clinical significance, current guidelines strongly recommend *DPYD* genotyping before initiating FP chemotherapy to mitigate the risk of life-threatening adverse reactions (13, 14).

Additional polymorphisms have been investigated for their potential influence on FP toxicity and efficacy; however, their impact is less pronounced, and routine genotyping for these variants is not currently recommended in clinical practice. These include *DPYD* polymorphisms such as DPYD*9 (c.85T>C *-* rs1801265*)*, and DPYD*5 (rs1801159), polymorphisms affecting the gene encoding 5-FU’s therapeutic target thymidylate synthase (*TYMS*) 5’-UTR VTNR 2R and 3R (rs45445694) and 3’-UTR + 1494 del6 (rs16430) variants, and polymorphisms affecting the methylenetetrahydrofolate reductase gene (*MTHFR*) variants c.677C>T (rs1801133) and c.1298A>C (rs1801131) (14–17).

**Figure 2:**
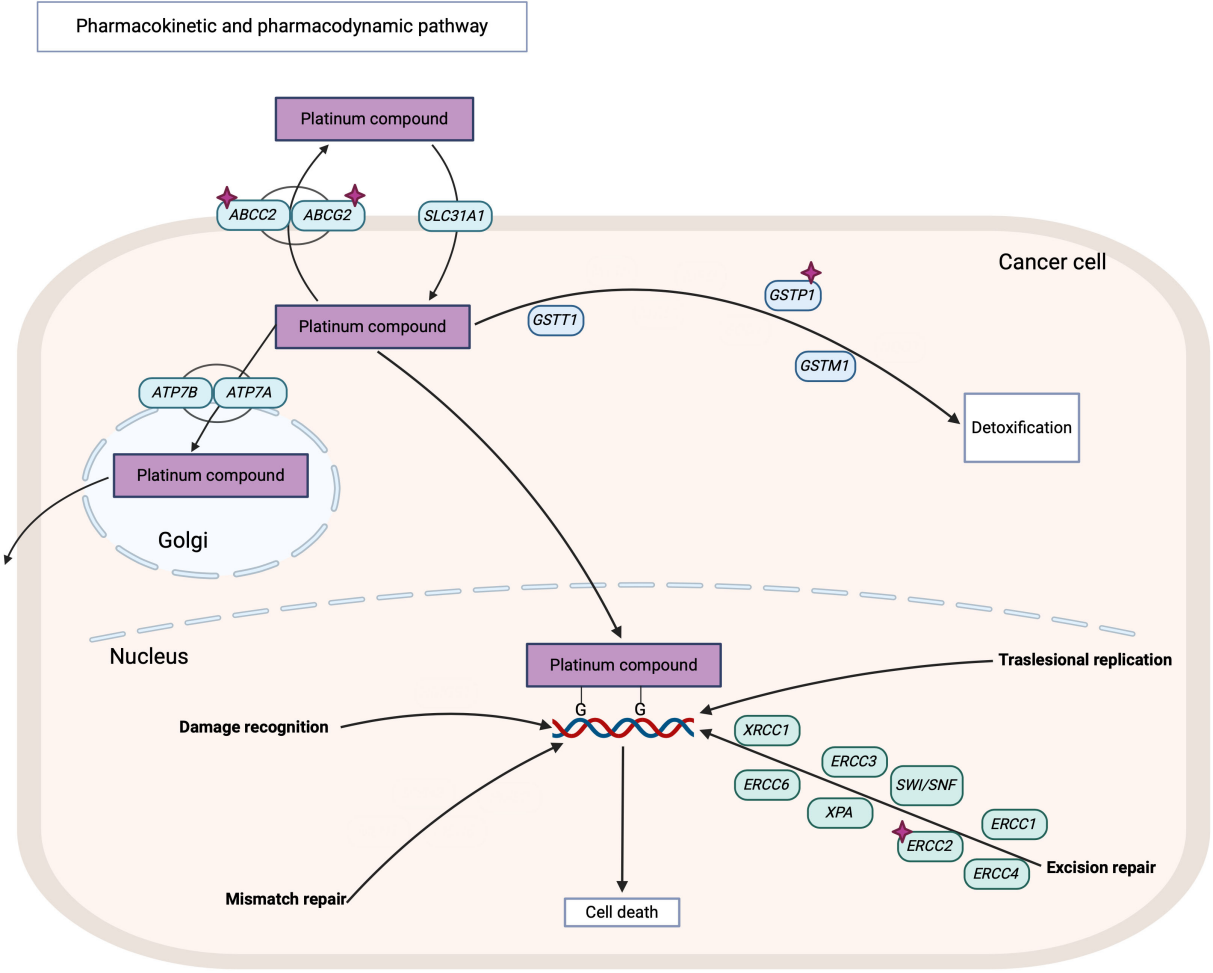
Pharmacokinetics and pharmacodynamics pathways of platinum compound including oxaliplatin. Purple stars indicate analyzed genes in this study. Image created with BioRender.com, adapted from PharmGKB.

On the other hand, L-OHP is an alkylating agent primarily eliminated through glutathione conjugation, a process mediated by the glutathione S-transferase (GST) family of enzymes (**Figure 2**). A genetic variant of the GST enzyme GSTP1 (rs1665 A>G) and the deletion of GSTM1 have been linked to peripheral neuropathy in patients undergoing modified FOLFOX6 treatment (18). Additionally, polymorphisms in the excision repair cross-complementation group 1 (ERCC1) and 2 (ERCC2) genes have been investigated in relation to L-OHP and FOLFOX toxicity, yielding conflicting results (18–20). Polymorphisms in the ABC transporters—*ABCB1* c.3435C>T (rs1045642), *ABCG2* c.421C>G (rs2231142), and *ABCC4* A>C/A>T (rs3742106)—have been associated with adverse clinical outcomes, including reduced survival and diminished response to FOLFOX and CAPOX treatment (21, 22).

Overall, the evidence suggests that genotyping genes involved in the metabolism of 5-FU, L-OHP, and other commonly used drugs in CRC chemotherapy may provide valuable insight into the factors contributing to ADRs in CRC patients. In the best-case scenario, this approach could serve as a useful tool for assessing ADR risk in advance.

Our research team has previously proposed similar predictive tools for testicular cancer (Lavanderos et al., 2019 (23)) and hematological cancers (Martinez et al., 2020 (24)). While both models require further validation before clinical implementation, they highlight the significant potential of pharmacogenetic tools in preventing ADRs in chemotherapy patients.

Therefore, this study aims to identify polymorphic variants of *TYMS, TYMP, DPYD, GSTP1, MTHFR, ERCC2*, and *ABC* genes to establish a predictive model for ADRs in advanced CRC patients.

## 2 Materials and methods

### 2.1 Pre-selection of genetic variants

Polymorphic variants of *TYMS, TYMP, DPYD, GSTP1, MTHFR, ERCC2*, and *ABC* genes were filtered to select the most relevant ones based on current clinical evidence. A score was given to the candidate polymorphism based on the level of clinical evidence according to the *PharmGKB* database (https://www.pharmgkb.org/). A second score was assigned based on the minor allele frequency reported in the *Ensemble* database (https://www.ensembl.org/). A third score was assigned based on the reported outcome of the genetic variant in terms of toxicity or progression-free survival in patients treated with FP-based chemotherapy. The fourth score was based on whether the variant caused a change in the protein’s amino acidic sequence, or on the contrary, it only affected non-coding regions of the gene (inter-intragenic and intronic regions) or was a synonymous change maintaining the protein’s original sequence. The last two scores were based on the predicted impact of amino acid substitution in the resulting protein function based on analysis of the protein sequence using the bioinformatic tools *PolyPhen* (http://genetics.bwh.harvard.edu/pph2/) and *SIFT* (https://sift.bii.a-star.edu.sg/) when possible (**Supplementary Table 1**) (25, 26).

### 2.2 Patients and clinical data

Advanced CRC patients who had previously consented to donate blood samples to the Biobank of Fluids and Tissues of the Clinical Hospital of the University of Chile (BTUCH), the National Cancer Institute (INC), and the Arturo López Pérez Foundation (FALP) were analyzed for this study. Inclusion criteria included: (1) patients older than 18 years diagnosed with advanced CRC (stage III or IV), (2) patients with complete clinical data available in the treating hospital, and (3) patients treated with FP-based chemotherapy (FOLFOX, FOLFIRI, CAPOX, Capecitabine) as the first line of treatment. Exclusion criteria included: (1) patients with histology other than adenocarcinoma, (2) no primary tumor specimen available, (3) included in other interventional clinical studies as part of metastatic CRC treatment, (4) patients presenting microsatellite instability, (5) patients who have abandoned their treatment for unknown reasons, (6) patients presenting secondary cancers whether or not they were under chemotherapeutic treatments, (7) patients treated only with biologic treatment as colorectal cancer therapy.

Clinical data was collected, including sex, age, date of diagnosis, date of death, date of first and second-line chemotherapy treatment and scheme used, anatomical pathology and medical oncology diagnosis, TNM stage, associated co-morbidities, smoking habit, alcoholic habit, ADRs within each recorded cycle, laboratory tests, imaging tests, among others. All the clinical data was documented into an eCRF (electronic case report form) on the RedCap^®^ platform and then compiled and categorized for further analysis, strictly following standardized BTUCH procedures to protect the patient’s sensitive data.

### 2.3 DNA extraction from blood samples

Genomic DNA was extracted from a sample of 6 mL of blood from patients using the E.Z.N.A.^®^ Blood DNA Mini Kit (Omega Bio-tek, Inc. Georgia, USA.) following manufacturer’s instructions. The concentration and integrity of the extracted DNA were assessed using a DeNovix DS-11 Series Spectrophotometer (DeNovix Inc. Delaware, USA.) and confirmed through 1.2% agarose gel electrophoresis. Samples were diluted to 10–20 ng/mL for further analysis.

### 2.4 Genotyping of selected genetic variants

Genetic variants were primarily genotyped using real-time-qPCR using *TaqMan*™ commercial probes in a Stratagene Mx3000p Real-Time PCR System and AriaMx Real-Time PCR System (Agilent Technologies, Santa Clara, CA, USA) (**Supplementary Table 2**). The *TYMS* rs45445694 variant was analyzed through PCR-RFLP using a G-Storm Thermocycler model GS00482 (G-Storm Ltd, Somerset, England), MyTaq™ DNA Polymerase and PCR Master Mix (Bioline*®* London, U.K.). The following primers were used: forward 5’-GCGGAAGGGGTCCTGCCA-3’ and *reverse* 5’-TCCGAGCCGGCCACAGGCAT-3’ (IDT Fermelo-Biotec, Chile). Genotypes were determined based on PCR product size, visualized on either a 2% agarose gel or an 18% polyacrylamide gel. A 102 bp product corresponded to the 2R/2R homozygous genotype, a 130 bp product to a 3R/3R homozygous genotype, and the presence of both bands indicated a heterozygous (2R/3R) genotype.

### 2.5 Statistical analysis

Patients were categorized into two groups: exposed (carriers of the risk allele) and non-exposed (carriers of the non-risk allele). The primary outcome was defined as the occurrence of any ADR and its severity, graded from 1 (less severe) to 4 (more severe) according to the Common Terminology Criteria for Adverse Events (CTCAE) guideline v5.0 (27).

Descriptive statistical analyses were conducted for all collected data using the measures of central tendency, including mean, median, and standard deviation (SD). The presence and severity of ADRs during the FP-based treatment were analyzed through logistic regression, with results expressed as the corresponding Odds Ratio (OR) and p-value. Genetic variants were assessed using co-dominant, recessive, and dominant inheritance models. Additionally, multivariate logistic regression analyses were performed using a stepwise selection method, with the optimal model chosen based on sensitivity, accuracy, and receiver operating characteristic (ROC) curves.

All statistical analyses were conducted using the R version 4.2.2 in RStudio version 2022.12.0 + 353. A statistical power of 80% and a significance level of 95% were considered for all analyses.

### 2.6 Ethical considerations

This study was approved by the Scientific Ethical Committee of the North Metropolitan Health Service and the Scientific Ethical Committee of the Faculty of Medicine of the University of Chile. It was conducted in accordance with the ethical principles outlined in the Declaration of Helsinki (1964), Good Clinical Practices (GCP), and Chilean National Laws N° 20.120, N° 19.628, and N° 20.584.

## 3 Results

### 3.1 Selection of genetic variants

To identify relevant genetic variants, we compiled a comprehensive database of variants associated with the response to 5-FU, L-OHP, and irinotecan from the PharmGKB database (https://www.pharmgkb.org/). Each variant was evaluated based on the criteria outlined in **Supplementary Table 1**, leading to the selection of 16 genetic variants for further analysis. These variants included: *TYMS* (rs4544694 and rs11280056), *TYMP* (rs11479), *DPYD* (rs6737679 and rs1801265), *GSTP1* (rs1695), *MTHFR* (rs1801131 and rs1801133), *ERCC2* (rs13181), *ABCB1* (rs1045642 and rs1128503), *ABCC2* (rs717620), *ABCC4* (rs9561778), and *ABCG2* (rs2231142).

### 3.2 Patient clinical characteristics

A total of 82 patients with advanced CRC were recruited from BTUCH, INC, and FALP for this study. The characteristics of the participants are summarized in **Table 1**. The sex distribution among participants was 58.5% male and 41.5% female. 14.6% of patients reported smoking habit, 21.9% reported to be ex-smokers (> 1 year) and 34.1% reported alcohol consumption. Most patients received FOLFOX or CAPOX as first-line chemotherapy, and 36.6% required a second-line chemotherapy regimen.

**Table 1:** Characteristics of patients.

Variable		
Sex	Percentage %	n
Male	58.5	48
Female	41.5	34
Age at diagnosis (yr)		
Average ± S.D.	60 ± 12.6	
Median	63	
Smoking habits	Percentage %	n
Smoker	14.6	12
Ex-smoker	21.9	18
Non-smoker	54.9	45
N.A.	8.5	7
Alcohol drinking habits	Percentage %	n
Drinker	34.1	28
Non-drinker	51.2	42
N.A.	14.6	12
First-line chemotherapy	Percentage %	n
5-FU/LV	1.2	1
Capecitabine	15.8	13
CAPOX	42.7	35
FOLFOX	31.7	26
FOLFOX + Cetuximab	2.4	2
FOLFOX + Bevacizumab	2.4	2
FOLFIRI	2.4	2
FOLFIRI + Cetuximab	1.2	1
Second-line chemotherapy	Percentage %	n
Yes	36.6	30
No	63.4	52
5-FU/LV	3.3	1
5-FU/LV + Cetuximab	6.7	2
Capecitabine	6.7	2
FOLFIRI	46.7	14
Capecitabine + Irinotecan	3.3	1
FOLFIRI + Aflibercept	3.3	1
FOLFIRI + Cetuximab	13.3	4
FOLFOX	10.0	3
FOLFOX + Bevacizumab	6.7	2

S.D., Standard deviation; N.A., Not available; n, Sample size; yr, Years; 5-FU/LV, 5-fluorouracil + leucovorin; CAPOX, capecitabine + oxaliplatin; FOLFOX, 5-fluorouracil + leucovorin + oxaliplatin; FOLFIRI, 5-fluorouracil + leucovorin + irinotecan.

### 3.3 Genotype frequencies of the selected genetic variants in the study population

Results of genotype frequencies are shown in **Table 2**. We initially considered analyse of *DPYD* SNPs c.1905 + 1G>A (DPYD*2) (rs3918290) and c.1679T>G (DPYD*13) (rs55886062). However, genotyping of both polymorphisms revealed no variability (DPYD*2 G/G genotype and DPYD*13 genotype T/T for all patients), and therefore both were omitted from the statistical analyses.

**Table 2:** Genotype frequencies of the selected genetic variants in the study population.

Gene variant	Genotype / percentage % (n)		
*TYMS* 3’UTR 6bp ins-del (rs11280056)	INS/INS	INS/DEL	DEL/DEL
	0.29 (24)	0.41 (34)	0.29 (24)
*TYMS* 5’UTR TSER (rs45445694)	2R/2R	2R/3R	3R/3R
	0.11 (9)	0.72 (59)	0.17 (14)
*TYMP* c.1412C>T (rs11479)	C/C	C/T	T/T
	0.76 (62)	0.20 (16)	0.04 (4)
*DPYD* c.1905+1G>A (DPYD*2) (rs3918290)	G/G	G/A	A/A
	1 (82)	0 (0)	0 (0)
*DPYD* c.1679T>G (DPYD*13) (rs55886062)	T/T	T/G	G/G
	1 (0)	0 (0)	0 (0)
*DPYD* c.2846A>T (rs67376798)	T/T	T/A	A/A
	0.98 (80)	0.01 (1)	0.01 (1)
*DPYD* c.85T>C (DPYD*9) (rs1801265)	T/T	T/C	C/C
	0.60 (49)	0.30 (25)	0.10 (8)
*GSTP1* c.313A>G (rs1695)	A/A	A/G	G/G
	0.43 (35)	0.39 (32)	0.18 (15)
*MTHFR* c.1409A>C (rs1801131)	A/A	A/C	C/C
	0.59 (48)	0.35 (29)	0.06 (5)
*MTHFR* c.788C>T (rs1801133)	C/C	C/T	T/T
	0.31 (25)	0.47 (39)	0.22 (18)
*ERCC2* c.2251A>C (rs13181)	A/A	A/C	C/C
	0.72 (59)	0.23 (19)	0.05 (4)
*ABCB1* c.3435T>C (rs1045642)	C/C	C/T	T/T
	0.33 (27)	0.51 (42)	0.16 (13)
*ABCB1* c.1236T>C (rs1128503)	T/T	T/C	C/C
	0.13 (11)	0.55 (45)	0.32 (26)
*ABCC2* c.-24C>T (rs717620)	C/C	C/T	T/T
	0.71 (58)	0.25 (21)	0.04 (3)
*ABCC4* c.3366+1243C>A (rs9561778)	C/C	C/A	A/A
	0.93 (76)	0.05 (4)	0.02 (2)
*ABCG2* c.421C>A (rs2231142)	C/C	C/A	A/A
	0.88 (72)	0.10 (8)	0.02 (2)

n, Sample size; UTR, Untranslated region; bp, base pair; ins, 6bp insertion; del, 6bp deletion; TSER, Thymidylate synthase enhancer region; 2R, 2 repeat tandem enhancer region; 3R, 3 repeat tandem enhancers regions.

### 3.4 ADR in the study population

We identified 54 types of ADR occurring during chemotherapy. After categorizing them according to the CTCAE guideline v5.0, we observed that most commonly affected system was the gastrointestinal, followed by the nervous system, and fatigue, pain, hematological disorders, skin and subcutaneous tissue disorders. Additionally, ADRs were classified by severity, with grades 0 to 2 considered non-severe and grades 3 to 4 considered severe. The frequency distribution of these classifications is presented in **Table 3**.

**Table 3:** Frequency of ADRs among patients.

Type of ADRs	Presence % (n)		*Severity % (n)	
	Yes	No	Not severe (Grade 0-2)	Severe (Grade 3-4)
Gastrointestinal disorders	82.9 (68)	17.1 (14)	84.1 (69)	15.9 (13)
Haematological disorders	40.2 (33)	59.8 (49)	85.4 (70)	14.6 (12)
Pain	46.3 (38)	53.7 (44)	95.1 (78)	4.9 (4)
Skin and subcutaneous tissue disorders	19.6 (11)	80.4 (71)	98.0 (50)	2.0 (1)
Fatigue	53.7 (44)	46.3 (38)	98.8 (81)	1 (1.2)
Nervous system disorders	74.5 (38)	25.5 (31)	92.2 (47)	7.8 (4)

ADRs, Adverse drug reactions; n, Sample size. * Common Terminology Criteria for Adverse Events (CTCAE) guideline v5.0.

Some remarkable ADR contained in these categories are the presence of gastrointestinal disorders, where mucositis occurred in 34.1% (n: 28) and vomiting in 29.4% (n: 24) of patients (data not shown). The presence of neuropathy, included in the nervous system disorders, occurred in 37.8% (n: 31) of patients. Finally, thrombocytopenia occurred in 17.1% (n: 14) and anemia affected in 20.7% (n: 17) of patients, both included in the hematological disorders category.

### 3.5 Univariate analysis of the correlation between genotypes and toxicities

The next stage of analysis involved evaluating the correlation between the polymorphisms in the *TYMS*, *TYMP*, *DPYD*, *GSTP1*, *MTHFR*, *ERCC2*, and *ABC* genes and the risk of ADR of any severity during FU-based chemotherapy. This was done using univariate logistic regression and applying different inheritance models (dominant, recessive, and codominant), with the results of these analyses shown in **Table 4**. We identified a protective correlation between the 6bp deletion of the *TYMS* 3’UTR region (rs11280056) and a lower risk of gastrointestinal and hematological disorders in chemotherapy patients. Additionally, the C/C genotype of the *ERCC2* rs13181 polymorphism was correlated with a lower risk of gastrointestinal disorders in our study population.

**Table 4:** Univariate analysis of the correlation between the risk of ADRs and genotypes.

ADR/Polymorphism / Model	Genotypes	OR	CI (95%)	p-value
Gastrointestinal disorders				
*TYMS* 3’UTR 6bp ins-del (rs11280056)				
Codominant	INS/INS	1.000	--	Ref.
	INS/DEL	0.152	0.007 – 0.991	0.093
	DEL/DEL	0.100	0.004 – 0.071	0.045*
*ERCC2* c.2251A>C (rs13181)				
Codominant	A/A	1.000	--	Ref.
	A/C	0.152	0.007 – 0.991	0.093
	C/C	0.100	0.004 – 0.710	0.045*
Hematological disorders				
*TYMS* 3’UTR 6bp ins-del (rs11280056)				
Codominant	INS/INS	1.000	--	Ref.
	INS/DEL	0.156	0.044 – 0.498	0.002*
	DEL/DEL	0.029	0.004 – 0.182	0.001*
Dominant	INS/INS	1.000	--	Ref.
	INS/DEL + DEL/DEL	0.107	0.030 – 0.303	<0.0001*
Recessive	INS/INS + INS/DEL	1.000	--	Ref.
	DEL/DEL	0.075	0.004 – 0.421	0.015*
*GSTP1* c.313A>G (rs1695)				
Dominant	A/A	1.000	--	Ref.
	A/G+G/G	0.298	0.013 – 0.617	0.001*
Recessive	A/A+A/G	1.000	--	Ref.
	G/G	0.157	0.048 – 0.414	0.0005*
Pain				
*TYMP* c.1412C>T (rs11479)				
Dominant	C/C	1.000	--	Ref.
	C/T+T/T	5.860	1.381 – 31.420	0.027*
*ABCB1* c.1236T>C (rs1128503)				
Recessive	T/T+T/C	1.000	--	Ref.
	C/C	4.200	1.038 – 19.256	0.049*
*MTHFR* c.1409A>C (rs1801131)				
Codominant	A/A	1.000	--	Ref.
	A/C	0.355	0.126 – 0.947	0.042*
	C/C	0.750	0.028 – 19.827	0.842
Dominant	A/A	1.000	--	Ref.
	A/C+C/C	0.375	0.137 – 0.976	0.048*
Skin and subcutaneous tissue disorders				
*TYMS* 5'UTR 2R3R (rs45445694)				
Recessive	2R/2R + 2R/3R	1.000	--	Ref.
	3R/3R	6.400	1.188 – 36.705	0.029*
Nervous system disorders				
*GSTP1* c.313A>G (rs1695)				
Codominant	A/A	1.000	--	Ref.
	A/G	0.352	0.045 – 1.906	0.251
	G/G	0.098	0.011 – 0.570	0.015*
Recessive	A/A+A/G	1.000	--	Ref.
	G/G	0.182	0.040 – 0.759	0.020*
*MTHFR* c.1409A>C (rs1801131)				
Dominant	A/A	1.000	--	Ref.
	A/C+C/C	5.805	1.324 – 40.992	0.035*

OR, Odds ratio; CI, Confidence interval; Ref., reference genotype; bp, base pair; ins, 6bp insertion; del, 6bp deletion; TSER, Thymidylate synthase enhancer region; 2R, 2 repeat tandem enhancer region; 3R, 3 repeat tandem enhancers regions. *p-value < 0.05 is considered statistically significant.

The G allele of the *GSTP1* rs1695 polymorphism was correlated with a lower risk of hematological and nervous system disorders in treated patients, including a reduced risk of neuropathy. This was observed through the analysis of individual ADR using univariate logistic regression without CTCAE v5.0 grouping (**Supplementary Table 3**, OR = 0.01, p-value = 0.008). However, we observed a higher risk of mucositis in patients carrying this polymorphism (**Supplementary Table 3**, OR = 2.27, p-value = 0.036).

Further analysis of ungrouped ADRs indicated a higher risk of neuropathy in patients carrying the C allele of the *DPYD* rs1801265 polymorphism (**Supplementary Table 3**, codominant model T/C genotype OR = 30.0, p-value = 0.0049, dominant model OR = 4.583, p-value = 0.05).

Additionally, we found a direct correlation between the T allele of the *TYMP* rs11479 polymorphism and an increased risk of pain in chemotherapy patients. Similarly, patients carrying the C/C genotype of the *ABCB1* rs1128503 polymorphism exhibited a higher risk of pain. Conversely, the C allele of the *MTHFR* rs1801131 polymorphism was correlated with a lower risk of pain, but a higher risk of nervous system disorders in chemotherapy patients. Lastly, the 3R/3R genotype of the *TYMS* rs45445694 polymorphism was associated with an increased risk of skin and subcutaneous tissue disorders. No correlation was found between the risk of grouped and ungrouped ADR, and the other polymorphisms considered in this study.

When analyzing the risk of grouped ADR while considering only severe reactions (Grade III-IV), we found that only the *TYMS* 3’UTR 6bp deletion (rs11280056) was the only polymorphism associated with a lower risk of severe hematological disorders (dominant model OR = 0,098, CI 95% 0.013 – 0.441, p-value =0.005).

### 3.6 Multivariate analysis of genotypes and toxicities

Based on the univariate analysis of selected polymorphisms and the risk of grouped and ungrouped ADRs, we filtered all polymorphisms that resulted in an association with a p-value < 0.02 for step-by-step multivariate logistic regression. Our goal was to develop a multivariate model with good specificity and accuracy, incorporating at least two genetic variants.

**Figure 3:**
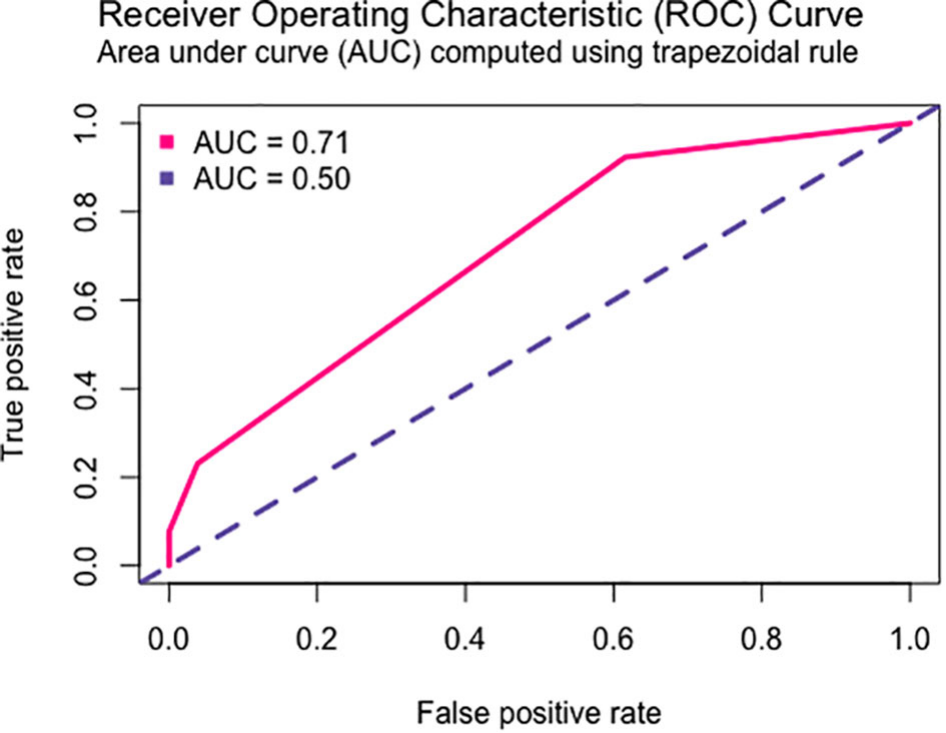
ROC curve of the multivariate model of risk of anemia in CRC patients.

Using the of *TYMP* rs11479 and *ABCB1* rs1044642 genetic polymorphisms, we generated the best predictive model for anemia (ungrouped ADR), applying a recessive inheritance model for both variants. The model is shown in **Table 5**, and the corresponding ROC curve is displayed in **Figure 3**.

**Table 5:** Multivariate model of the correlation between the *TYMP* rs11479 and *ABCB1* rs1044642 genotypes and the risk of anemia.

Variable		β	SE	OR	CI (95%)	p-value
Intercept		-3.667	1.602	0.026	0.001 - 0.428	0.022
*ABCB1* (rs1045642) T/T		-1.877	1.119	0.153	0.007 - 0.975	0.093
*TYMP* (rs11479) T/T		3.168	1.53	23.760	1.722 - 986.648	0.038*
Significancy and accuracy parameters of the model						
χ²	Degrees of freedom	Model’s p-value	Pseudo-R^2^	Accuracy	Sensitivity	AIC
7.22	2	0.027*	0.145	71.795	0.231	48.422

SE, Standard error; OR, Odds ratio; CI, Confidence interval; χ², Chi-squared test; AIC, Akaike information criterion. *p-value < 0.05 is considered statistically significant.

**Figure 4:**
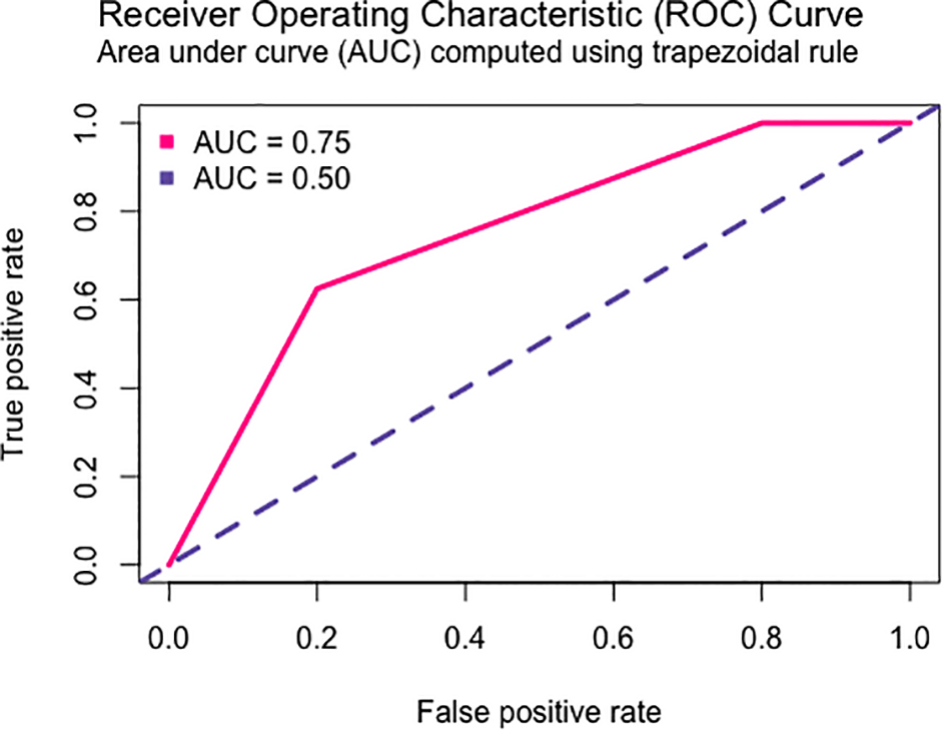
ROC curve of the multivariate model of risk of pain in CRC patients receiving FU-based chemotherapy.

For the ADR category pain, we obtained the best multivariate risk model with the *MTHFR* rs180131 (dominant model) and the *ERCC2* rs13181 polymorphism (recessive model). The resulting model and the model’s ROC curve are shown in **Table 6** and **Figure 4**.

**Table 6:** Multivariate model of the correlation between the *MTHFR rs180131 and ERCC2 rs13181* genotypes and *the risk of pain.*.

Variable		β	SE	OR	CI (95%)	p-value
Intercept		-2.663	1.45	0.026	0.003 - 0.999	0.066
*ERCC2* (rs13181) C/C		1.836	0.864	6.272	1.285 - 40.539	0.034*
*MTHFR* (rs1801131) A/C+C/C		-1.836	0.864	0.159	0.025 - 0.778	0.034*
Significancy and accuracy parameters of the model						
χ²	Degrees of freedom	Model’s p-value	Pseudo-R^2^	Accuracy	Sensitivity	AIC
9.161	2	0.01*	0.185	72.222	0.625	0.8

SE, Standard error; OR, Odds ratio; CI, Confidence interval; χ², Chi-squared test; AIC, Akaike information criterion. *p-value < 0.05 is considered statistically significant.

Unfortunately, we were unable to generate a multivariate risk model that met the minimum level of statistical significance for the remaining grouped and ungrouped ADR and selected polymorphisms.

## 4 Discussion

Despite the life-threatening risk posed patients with DPD deficiency (28), in Chile genotyping of *DPYD* genetic variants is still not recommended by the national clinical guidelines for CRC management or for other cancers that require FP-based chemotherapy schemes. Thus, in this study, we aimed to establish a correlation between the patient’s genotype and the risk of ADR in Chilean advanced CRC patients. Similarly, another Chilean study demonstrated that certain *DPYD* genetic variants, not included in this study, are associated with 5-FU toxicities, either in combination with L-OHP individually or alongside *UMPS* (rs1801019) and *ABCC2* (rs717620) genetic variants (29).

In this study, we included the *DPYD* genetic variant rs1801265, which, unlike other *DPYD* variants, is present in 26% of the world population (30). The C allele was identified as a risk factor for the development of neuropathies. The association of this genetic variant and ADR risk has been described in some recent studies (31), with evidence suggesting that analyzing genetic variants not associated with enzyme loss-of-function, such as rs1801265, can provide insights into DPD enzyme function and the risk of developing ADR in 5-FU treatment’s (32). These studies recommend haplotype analysis or exome sequencing, which could improve the understanding of ADR risk and DPD enzyme function.

The main therapeutic target of 5-FU is the inhibition of the TS enzyme (**Figure 1**). Among the key genetic variants, the 6bp deletion in the 3’UTR (rs11280056) was associated with a decreased risk of gastrointestinal and hematological ADRs. These findings contrast with previous studies, which reported that this polymorphism, along with *TYMS* 5’UTR TSER (rs45445694) is associated with increased severity of hematological ADR. However, those studies were based on a small sample size (6 patients with ADR) (33). This same genetic variant has also been linked to an increased risk of peripheral neuropathy (34) and severe hand-foot-syndrome (35). Additionally, 5-FU exerts a secondary effect by covalently binding to DNA and RNA, thereby inhibiting cellular replication and transcription. Similarly, L-OHP acts directly by binding to DNA, preventing these processes. At this stage, DNA repair systems play a crucial role, as their inhibition can influence ADR occurrence. Several SNPs in key DNA-repair genes, such as *ERCC2* can impact these pathways. One of the most studied SNPs in this research is rs13181 (c.2251A>C), which causes structural alterations in the protein, affecting its DNA-binding ability. In this study we observed that the CC genotype was a protective factor against gastric ADR. However, this result contradicts previous studies, where the CC genotype was linked to a reduction in chemotherapy dose requirements (36). Other studies have failed to find an association between this genotype and ADR risk (37). These discrepancies may be explained by mRNA overexpression as a compensatory mechanism for decreased enzyme functionality, a phenomenon previously described for ERCC1 in relation to survival (38). *In vitro* studies are needed to validate this hypothesis.

Within 5-FU pharmacokinetics, thymidate phosphorylase (TP) is a key enzyme responsible for initiating the activation of 5-FU into its active metabolite, which inhibits TS. Additionally, TP is crucial for the biotransformation of capecitabine (5-FU pro-drug). The *TYMP* c.1412C>T (rs11479) genetic variant, is a missense mutation that induces a structural change in the protein, affecting its catalytic activity. In this study it was observed that the T allele was associated with pain development. This same variant has previously been linked to early development of ADR onset in CRC patients undergoing fluoropyrimidine treatment (39).

For 5-FU metabolites to effectively inhibit TS, the folate cycle must be functional, as it generates TS’s secondary substrate. Without this substrate, TS is unable to bind FdUMP preventing its inhibitory activity (**Figure 1**). The MTHFR enzyme plays a crucial role in this process, as it catalyzes the conversion of 5,10-MTHF to 5-methyltetrahydrofolate (5-MTHF) (40). The *MTHFR* rs1801133 (c.788C>G) genetic variant induces a protein conformational change, reducing enzyme activity, which may contribute to ADR risk in this treatment (41). In our study, the C allele was associated with an increased risk of nervous system ADR, yet it also acted as a protective factor against pain development. These findings have not been previously reported. Past research has only linked this variant to asthenia (42), mucositis, diarrhea and neutropenia (43).

L-OHP is an alkylating agent primarily eliminated through glutathione conjugation, mediated by glutathione S-transferase (GST) enzymes (**Figure 2**). One of the most studied SNPs associated with CRC treatment-related ADR is *GSTP1* rs1695 (c.313A>G), which alters protein structure (44). Our findings indicate that the A allele is associated with an increased risk of hematological and nervous system ADR, aligning with previous studies (45). Interestingly, we also observed that the G allele was associated with a higher incidence of mucositis in this cohort, a novel finding that has not been reported before.

Regarding multivariate models, only two models were successfully generated, one predicting anemia risk and another predicting pain risk. In the study by Deng et al., 2020, *DPYD* and *GSTP1* were identified has strong predictors of ADR, including anemia (46). In contrast, our anemia predictor model incorporated *ABCB1* and *TYMP* variants, which have not been previously associated with anemia risk. However, it is important to note that the *ABCB1* variant was not statistically significant within the model. Previous studies have linked anemia risk solely to *DPYD* variants (47), unlike the finding of this study.

These findings emphasize the importance of considering population-specific genetic variability in cancer pharmacogenomic analyses. However, the relatively small sample size, gaps in clinical records, and lack of longitudinal follow-up limit the broader applicability of our results. Future research should prioritize larger, multiethnic cohorts—particularly from European populations—to validate these findings and further the development of personalized medicine.

## 5 Conclusion

This study provides a foundation for developing pharmacogenetic-based predictive models for adverse reactions associated with 5-FU, including nervous system disorders, mucositis, and hematological and skin toxicities. Future research may refine these models to enable personalized dose adjustments, improving chemotherapy safety in Chilean colorectal patients.

## Data Availability

Publicly available datasets were analyzed in this study. This data can be found here: https://figshare.com/s/4c690e15483ef6f20ba8.
