# Supplementary material for: Genetic variants and clinical determinants affecting the response to 5-Fluorouracil-based treatment in Chilean patients with advanced colorectal cancer
Source: Front Oncol. 2025 Jul 25;15:1589724. doi: 10.3389/fonc.2025.1589724 (PMC12331468; doi:10.3389/fonc.2025.1589724)
Supplement: Supplementary file 1 [file Table1.docx]

Supplementary Material

# Supplementary Data

Supplementary Table 1. Scores used as selection criteria for genetic variants

| **Criteria** |  | **Assigned Score** |
| --- | --- | --- |
| Level of clinical evidence (PharmGKB) | Level 1 | 4 |
|  | Level 2 | 3 |
|  | Level 3 | 2 |
|  | Level 4 | 1 |
| MAF | 0.01 – 0.25 | 1 |
|  | 0.26 – 0.50 | 2 |
|  | 0.51 – 0.75 | 3 |
|  | 0.76 – 0.99 | 4 |
| Relation between the variant and the outcome | Corresponds | 2 |
|  | Does not correspond | 1 |
| Effect of the variant on the protein sequence | Amino acid change | 4 |
|  | Intergenic, intron or silent change | 2 |
| *PolyPhen* | Benign | 1 |
|  | Malign | 2 |
| *SIFT* | Tolerated | 0.5 |
|  | Harmful | 1 |

*MAF: minor allele frequency. SIFT: Sorting Intolerant From Tolerant. PolyPhen: Polymorphism Phenotyping.*

Supplementary Table 2. *TaqMan*™ probes used in genotypification of genetic variants

| **Gene** | **SNP** | **Genomic change** | **TaqMan™ ID** | ***TaqMan*™ Context Sequence** |
| --- | --- | --- | --- | --- |
| *ABCB1* | rs1045642 | c.3435T>C | C___7586657_20 | TGTTGGCCTCCTTTGCTGCCCTCAC**[A/G]**ATCTCTTCCTGTGACACCACCCGGC |
|  | rs1128503 | c.1236T>C | C___7586662_10 | GCCCACTCTGCACCTTCAGGTTCAG**[A/G]**CCCTTCAAGATCTACCAGGACGAGT |
| *ABCC2* | rs717620 | c.-24C>T (5`UTR) | C___2814642_10 | ACAATCATATTAATAGAAGAGTCTT**[C/T]**GTTCCAGACGCAGTCCAGGAATCAT |
| *ABCC4* | rs9561778 | c.3366++1243C>A | C__31356326_10 | AAAAATCACATTCTCCTTCCCTTCC**[G/T]**GTGGCACGTTCTCTATGCTTCCTAC |
| *ABCG2* | rs2231142 | c.421C>A | C__15854163_70 | GCAAGCCGAAGAGCTGCTGAGAACT**[G/T]**TAAGTTTTCTCTCACCGTCAGAGTG |
| *ERCC2* | rs13181 | c.2251A>C | C___3145033_10 | TGCTGAGCAATCTGCTCTATCCTCT**[G/T]**CAGCGTCTCCTCTGATTCTAGCTGC |
| *DPYD* | rs3918290 | c.1905+1G>A (*DPYD*2*) | [C__30633851_20](https://www.thermofisher.com/order/genome-database/details/genotyping/C__30633851_20) | TGTTTTAGATGTTAAATCACACTTA**[C/T]**GTTGTCTGGAAAGTCAGCCTTTAGT |
|  | rs55886062 | c.1679T>G (*DPYD*13*) | [C__11985548_10](https://www.thermofisher.com/order/genome-database/details/genotyping/C__11985548_10) | CCATCCAGCTTCAAAAGCTCTTCGA**[A/C]**TCATTGATGTGCTGGTGGCTGGAGT |
|  | rs67376798 | c.2846A>T | C__27530948_10 | ACCACAGTTGATACACATTTCTTCA**[A/T]**CAATCATAGCCACAACTTGCTCTAC |
|  | rs1801265 | c.85T>C | C___9491497_10 | TCTAATTTCTTGGCCGAAGTGGAAC**[A/G]**CAGAGTTGCATGAGTTTGTGTTCGA |
| *GSTP1* | rs1695 | c.313A>G | C___3237198_20 | CGTGGAGGACCTCCGCTGCAAATAC**[A/G]**TCTCCCTCATCTACACCAACTATGT |
| *TYMP* | rs11479 | c.1412C>T | C__11946264_20 | CGGCAGAACGAGCTCTGCGAAGGGC**[A/G]**AGGGGGCGGCGAATGGCGCGCGGTC |
| *TYMS* | rs11280056 | c.*456_*455insAAGTTA | C_165213789_20 | ATGTAGAGTGTGGTTATGAACT**[TTAAAG/~]**TTATAGTTGTTTTATATGTTGCT |
| *MTHFR* | rs1801131 | c.1409A>C | C____850486_20 | AAGAACGAAGACTTCAAAGACACTT**[G/T]**CTTCACTGGTCAGCTCCTCCCCCCA |
|  | rs1801133 | c.665C>G | C___1202883_20 | GAAAAGCTGCGTGATGATGAAATCG**[G/A]**CTCCCGCAGACACCTTCTCCTTCAA |

*The nucleotide change that causes the polymorphism is highlighted in bold and between brackets. “~”: Deletion.*

Supplemental Table 3. Univariate analysis of the correlation between the risk of non-grouped ADRs and genotypes

| **ADR / Polymorphism / Model** | **Genotype** | **OR** | **CI (95%)** | **p-value** |
| --- | --- | --- | --- | --- |
| **Neuropathy** |  |  |  |  |
| *GSTP1* c.313A>G (rs1695) |  |  |  |  |
| Codominant | A/A | 1.000 | -- | Ref. |
|  | A/G | 0.495 | 0.108 – 2.053 | 0.337 |
|  | G/G | 0.100 | 0.015 – 0.509 | **0.008*** |
| Recessive | A/A+A/G | 1.000 | -- | Ref. |
|  | G/G | 0.147 | 0.028 – 0.610 | **0.012*** |
| *DPYD* c.85T>C (rs1801265) |  |  |  |  |
| Codominant | T/T | 1.000 | -- | Ref. |
|  | T/C | 30.000 | 3.994 – 649.215 | **0.0049*** |
|  | C/C | 1.000 | 0.042 – 10.846 | 1 |
| Dominant | T/T | 1.000 | -- | Ref. |
|  | T/C+C/C | 4.583 | 1.051 – 23.335 | **0.05*** |
| **Mucositis** |  |  |  |  |
| *GSTP1* c.313A>G (rs1695) |  |  |  |  |
| Dominant | A/A | 1.000 | -- | Ref. |
|  | A/G+G/G | 2.270 | 1.090 – 5.148 | **0.036*** |

*ADR: Adverse drug reaction. OR: Odds ratio. CI: Confidence interval. Ref.: reference genotype. A p-value < 0.05 is highlighted and considered statistically significant.*
